# The CDK7 inhibitor CT7001 (Samuraciclib) targets proliferation pathways to inhibit advanced prostate cancer

**DOI:** 10.1038/s41416-023-02252-8

**Published:** 2023-04-19

**Authors:** Theodora A. Constantin, Anabel Varela-Carver, Kyle K. Greenland, Gilberto Serrano de Almeida, Ellen Olden, Lucy Penfold, Simon Ang, Alice Ormrod, Damien A. Leach, Chun-Fui Lai, Edward K. Ainscow, Ash K. Bahl, David Carling, Matthew J. Fuchter, Simak Ali, Charlotte L. Bevan

**Affiliations:** 1 Imperial Centre for Translational and Experimental Medicine, Department of Surgery and Cancer, Imperial College London, Hammersmith Hospital Campus, London, UK; 2 MRC London Institute of Medical Sciences, Imperial College London, Hammersmith Hospital, London, UK; 3 Carrick Therapeutics, Nova UCD, Bellfield Innovation Park, Dublin, 4 Ireland; 4 Department of Chemistry, Molecular Sciences Research Hub, Imperial College London, White City Campus, London, UK

**Keywords:** Prostate cancer, Molecular biology

## Abstract

**Background:**

Current strategies to inhibit androgen receptor (AR) are circumvented in castration-resistant prostate cancer (CRPC). Cyclin-dependent kinase 7 (CDK7) promotes AR signalling, in addition to established roles in cell cycle and global transcription, providing a rationale for its therapeutic targeting in CRPC.

**Methods:**

The antitumour activity of CT7001, an orally bioavailable CDK7 inhibitor, was investigated across CRPC models in vitro and in xenograft models in vivo. Cell-based assays and transcriptomic analyses of treated xenografts were employed to investigate the mechanisms driving CT7001 activity, alone and in combination with the antiandrogen enzalutamide.

**Results:**

CT7001 selectively engages with CDK7 in prostate cancer cells, causing inhibition of proliferation and cell cycle arrest. Activation of p53, induction of apoptosis, and suppression of transcription mediated by full-length and constitutively active AR splice variants contribute to antitumour efficacy in vitro. Oral administration of CT7001 represses growth of CRPC xenografts and significantly augments growth inhibition achieved by enzalutamide. Transcriptome analyses of treated xenografts indicate cell cycle and AR inhibition as the mode of action of CT7001 in vivo.

**Conclusions:**

This study supports CDK7 inhibition as a strategy to target deregulated cell proliferation and demonstrates CT7001 is a promising CRPC therapeutic, alone or in combination with AR-targeting compounds.

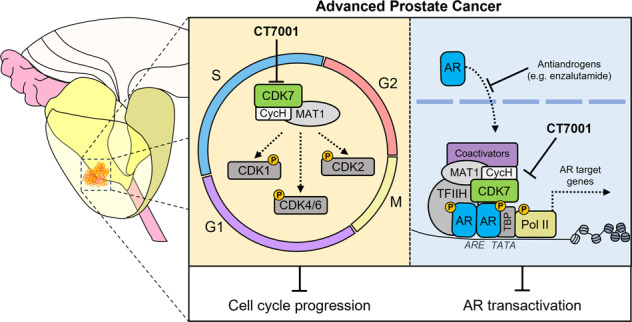

## Background

Prostate cancer is the second most common malignancy in men worldwide and a leading cause of cancer-related death [1]. The androgen receptor (AR) is a ligand-dependent nuclear receptor that plays a critical role in prostate cancer initiation and progression [2]. Therapies aiming to ablate AR activity by preventing ligand-dependent receptor activation are initially effective but circumvented following progression to a therapy-resistant stage termed castration-resistant prostate cancer (CRPC). Commonly, castration resistance mechanisms facilitate reactivation of the AR signalling pathway and downstream transcriptional programmes [3–5]. One phenomenon associated with disease progression, the expression of AR splice variants (AR-V), has drawn interest as a potential mechanism mediating androgen independency in CRPC [6–8]. AR-Vs lack the AR ligand-binding domain (LBD) but retain the N-terminal activation function, and thereby have the potential to mediate AR signalling while being insensitive to approved AR-targeted therapies, all of which target the LBD [9]. Novel therapeutic approaches are therefore needed for the treatment of CRPC, with strategies that interfere with oncogenic AR transcription in a LBD-independent manner being of particular interest [10].

Cyclin-dependent kinase 7 (CDK7) is a recognised anticancer drug target due to its regulatory roles in cell division and transcription [11, 12]. As part of the CDK-activating kinase (CAK) complex together with cyclin H and MAT1, CDK7 phosphorylates the T-loop of cell cycle CDKs, including CDK1, -2, -4 and -6, stimulating their activities in a temporal manner to drive progression through the cell cycle [13–16]. CAK also functions as a component of the general transcription factor TFIIH, enabling transcription initiation by phosphorylating serine-5 residues (S5) in the RNA polymerase II (PolII) C-terminal domain (CTD) [17]. Although phosphorylation of PolII CTD is essential for mRNA synthesis [18], whether CDK7 activity is strictly required for basal transcription is still debated [19–22]. It has previously been shown that CDK7 phosphorylates AR at serine-515 (S515) to regulate transactivation and proteasomal degradation of AR, and facilitates assembly of a transcriptionally active AR-coregulator complex [23–25]. Thus, CDK7 may be promoting oncogenic AR transcription in CRPC by both AR-specific and more global transcriptional effects. In line with a tumour-promoting role in CRPC, high tumour CDK7 protein levels are associated with faster biochemical recurrence, marked by rises in serum prostate-specific antigen (PSA) [26].

The multiple roles of CDK7 commend it as a drug target for CRPC. However, a fundamental understanding of the mechanistic effects of selective CDK7 inhibition in CRPC is currently lacking. In a previous study, the covalent CDK7 inhibitor THZ1 was used [23], however, this also targets CDK12/13, which substantially obscures the contribution of CDK7 inhibition to its downstream effects [21, 22, 27]. CT7001/Samuraciclib (formerly ICEC0942) is a novel, orally bioavailable, ATP-competitive inhibitor of CDK7 with preclinical activity in models of breast cancer, colon cancer, and acute myeloid leukaemia [28–32]. CT7001 is currently in phase I/II clinical trials for advanced solid malignancies (NCT03363893). Recently released data from this study demonstrated acceptable safety profile and evidence of antitumour activity, including PSA reductions in 4 CRPC patients [33, 34]. As CT7001 is currently undergoing clinical evaluation, a more comprehensive investigation of its preclinical activity in prostate cancer is warranted. Here, we describe the mechanism of tumour inhibition achieved by CT7001 in prostate cancer models in vitro and in vivo and explore its efficacy as monotherapy and in combination with the widely used AR antagonist enzalutamide.

## Materials and methods

### Chemicals

CT7001 was provided by Carrick Therapeutics. Apalutamide, enzalutamide, darolutamide and bavdegalutamide were purchased from MedChemExpress. Mibolerone was purchased from PerkinElmer.

### Cell lines

LNCaP, C4-2, VCaP, DU145, PC3, PNT1A, BPH-1 cell lines were obtained from the ATCC as frozen stocks. 22Rv1 and 22Rv1 FL-AR KO were a gift from Dr Luke Gaughan (Newcastle University) [35]. C4-2B cells were a gift from Prof Ian Mills (Oxford University) [36]. LNCaP/Luc cells are stably transfected with an AR-specific luciferase reporter construct [37] and selection was maintained with 500 µg/mL G418 (Sigma) and 10 µg/mL blasticidin (Melford Biolaboratories). All cell lines were cultured according to ATCC recommendations in growth medium with 10% fetal calf serum (FCS) and 2 mM L-glutamine at 37 °C in humidified incubators maintained at 5% CO_2_. Cell line authenticity was confirmed using short tandem repeat analysis performed through the MWG Eurofins Human Cell Line Authentication service. All cell lines were regularly tested for Mycoplasma infection using the MycoAlert Detection Kit (Lonza).

### Cellular thermal shift assay (CeTSA) in intact LNCaP cells

The melting temperatures (*T*_m_) of CDK targets were determined using a Boltzmann sigmoidal curve (least squares fit) fitted through the average normalised data points from all human lines available in the Meltome Atlas [38]. The melt curve for CDK7 was generated as previously described [39]. Isothermal dose-response fingerprints were generated at 54 °C as previously described [39] from intact LNCaP cells treated with dimethylsulfoxide (DMSO) or CT7001 (concentrations range 0.078–20 μM) for 3 h.

### Protein extraction

Following incubation with fresh growth medium containing CT7001, cells were harvested and lysed as previously described [37]. Protein content was measured using the Pierce BCA Protein Assay Kit (ThermoFisher).

### Immunoblotting

Immunoblotting was carried out as previously described [40] using the following primary antibodies: CDK7 (Cell Signaling Technology, 2916), CDK1 (Cell Signaling Technology, 9116), CDK2 (Cell Signaling Technology, 2546), CDK4 (Cell Signaling Technology, 12790), CDK9 (Cell Signaling Technology, 2316), β-actin (Abcam, ab6276), GAPDH (Cell Signaling Technology, 2118), P-S5 PolII (Abcam, ab5401), P-S2 PolII (Abcam, ab5095), PolII (Abcam, ab817), P-S780 Rb (Abcam, ab47763), P-S807/811 Rb (Cell Signaling Technology, 8516), Rb (Abcam, ab6075), cyclin H (Abcam, ab54903), MAT1 (Santa Cruz Biotechnology, sc135981), P-S15 p53 (Cell Signaling Technology, 9284), p53 (Santa Cruz Biotechnology, sc-126), P-T161/T160 CDK1/CDK2 (Abcam, ab201008), P-S10 Histone H3 (Abcam, ab139417), AR (MilliporeSigma, 06-680), P-T1457 MED1 (Abcam, ab60950), MED1 (Bethyl Laboratories, A300-793A), cleaved PARP1 (Abcam, ab4830), p21 (Santa Cruz Biotechnology, sc-817).

### Cell growth and drug synergy assays

Cell number was assayed using the Sulforhodamine B (SRB) assay as previously described [41]. To determine growth rate (GR) values for each treatment and derive GR metrics, a file containing cell number data from day 0 and day 3 was uploaded on the GRcalculator platform (www.grcalculator.org/grcalculator) [42].

CT7001 combinations with AR-targeted therapies were tested in a 6 × 5 matrix in LNCaP, C4-2B, and PC3 cells. Cell numbers on days 0 and 3 were assayed using the SRB assay. Percent growth inhibition was calculated relative to vehicle control using day 3 values corrected by subtracting day 0 values. A file containing growth inhibition (%) data for each cell line was uploaded on the SynergyFinder 2.0 web application (synergyfinder.fimm.fi) [43]. Bliss independence scores were calculated using default parameters.

### Caspase 3/7 assay

Caspase 3/7 activation assays were performed using the Caspase-Glo 3/7 Assay System (Promega), 72 h after treatment with CT7001. Luminescence was measured using the VICTOR Light luminometer (PerkinElmer). Signal was normalised to cell number on day 3 measured by SRB assay.

### RT-qPCR and TaqMan Low Density Array

Total RNA was extracted from treated cells using the RNeasy Mini kit (QIAGEN). cDNA was synthesised from 1 µg of RNA with oligo-dT primers using RevertAid First Strand cDNA Synthesis Kit (ThermoFisher). Quantitative Polymerase Chain Reaction (qPCR) was performed in a QuantStudio 7 Flex Real-Time PCR System (Applied Biosystems) using SYBR Green Real-Time PCR Master mix (Invitrogen) and primers designed using Primer-BLAST (listed in Additional File 2, Supplementary Table 1). Specificity was validated using melt curve analysis. Gene expression was normalised to RPL19, GAPDH and BACTIN and data were analysed using the ΔΔCt method. TaqMan Low-Density Array microfluidic cards were used to assay the expression of 32 AR targets (a list of the TaqMan qPCR assays is provided in Additional File 2, Supplementary Table 2). 22Rv1 FL-AR KO cells were treated 1 µM CT7001 in androgen-depleted medium (phenol red-free RPMI-1640 with 5% charcoal-stripped FCS). LNCaP and VCaP cells were treated with 1 µM CT7001 in androgen-repleted medium (androgen-depleted medium supplemented with 10 nM mibolerone). Total RNA was extracted after 24 h, and cDNA was synthesised as above. cDNA was mixed with TaqMan Gene Expression Master Mix (ThermoFisher) and loaded on the microfluidic card. Gene expression was normalised to GAPDH, RPLP0, TBP, and 18S and the data were analysed using the ΔΔCt method.

### Cell cycle analysis

Asynchronous cells treated with DMSO or CT7001 for 72 h were harvested and fixed in 70% ice-cold ethanol overnight. Fixed cells were washed twice with PBS, stained with the Muse Cell Cycle Reagent (Luminex), and analysed using the Guava Muse Cell Analyser (Luminex).

### Plasmids and site-directed mutagenesis

The following plasmids have been previously described: pSV-AR encoding human AR, TAT-GRE-E1B-LUC encoding AR-responsive luciferase reporter, and BOS-β-galactosidase encoding a constitutive reporter [44]. Plasmid constructs harbouring AR splice variants (pCMV5-CE1 encoding AR-V1, pCMV5-CE2 encoding AR-V6, pCMV5-CE3 encoding AR-V7, pCMV5-1/2/2b encoding AR-V3, pCMV5-1/2/3b encoding AR-V4, and pCMV5-v567es encoding AR-V567ES/AR-V12) were provided by Prof Scott Dehm (The University of Minnesota) [45]. pCMV5-hAR encoding full-length AR (FL-AR) was acquired from Addgene (plasmid #89078). The pSV-S515A-AR, pSV-S515E-AR, pSV-S515D-AR plasmids were synthesised from the pSV-AR template using the QuikChange Lightning Multi Site-directed mutagenesis kit (Agilent Technologies). Mutations were introduced using the following primers: 5’-GCAGAGTGCCCTATCCCGCTCCCACTTGTGTCAAAA-3’ for pSV-S515A-AR, 5’-GCAGAGTGCCCTATCCCGAGCCCACTTGTGTCAAAA-3’ for pSV-S515E-AR, and 5’-GCAGAGTGCCCTATCCCGATCCCACTTGTGTCAAAA-3’ for pSV-S515D-AR. Competent DH5α *E. coli* cells were transformed with the plasmids, plasmid DNAs were purified using QIAprep Spin Miniprep Kit (QIAGEN) and subjected to Sanger sequencing (GeneWiz) to confirm the presence of the expected mutations.

### Calcium phosphate-mediated transfections

COS-1 cells were transfected as previously described [44] with 1 μg luciferase reporter (TAT-GRE-E1B-LUC), 50 ng AR expression vector or empty vector and 50 ng BOS-β-galactosidase per well. Cells were incubated for 24 h in medium containing drug treatments.

### Luciferase and β-galactosidase assays

LNCaP/Luc or transfected COS-1 cells were lysed with Reporter Lysis Buffer (Promega). Luciferase activity was assayed using the Steadylite Plus kit (PerkinElmer). β-galactosidase activity was assayed using the Galacto-Light Plus system (Invitrogen). Light emission was measured using a VICTOR Light luminometer (PerkinElmer). For LNCaP/Luc lysates, luciferase activity was normalised to protein concentration. For COS-1 lysates, luciferase activity was normalised to β-galactosidase activity.

### C4-2B human CRPC xenografts

C4-2B cells were expanded by passaging twice weekly and harvested during the exponential growth phase. Cells were counted and viability was assessed using the trypan blue exclusion assay before implantation. Based on a priori sample size calculation using G*Power 3 software, a total of 40 male NSG mice (6-8-week-old) were purchased from Charles River (UK) and allowed to acclimatise for 1 week. Mice were injected subcutaneously with 2.5 × 10^6^ C4-2B cells resuspended in a volume of 100 µL serum-free media and Matrigel Basement Membrane Matrix High Concentration (Corning) in a 1:1 ratio. Animals were randomised into treatment groups (*n* = 10 per group) when tumour volume reached 90 mm^3^. Mice initiated on specific treatments were housed in separate cages. Individual mice were treated at 4 mL/kg body weight with vehicle (5% DMSO and 30% SBE-β-CD dissolved in distilled water), 50 mg/kg CT7001, 25 mg/kg enzalutamide, or 50 + 25 mg/kg CT7001 + enzalutamide (combination). Treatments were administered orally once daily for 21 days by two unblinded investigators. Tumour size was measured every 3–4 days using digital calipers by two unblinded investigators, and tumour volumes were calculated using the formula: length × width × height × *π*/6. Mice were sacrificed on the last day of treatment, 2 h after administration of the final dose.

### Mouse plasma assays

Whole blood was collected via cardiac puncture in EDTA-coated tubes and spun immediately at 7500 rpm for 5 min in a benchtop centrifuge. Plasma was collected, snap frozen in liquid nitrogen and sent to the Cambridge Biochemistry Assay Lab (Cambridge University Hospitals NHS Foundation Trust) for analysis. Mouse aspartate transaminase (AST), mouse urea, and human free PSA levels were measured using a Siemens Dimension EXL analyser.

### Immunohistochemistry

Dissected tumours were fixed in 4% paraformaldehyde for 48 h and embedded in paraffin blocks. Sections of 4 µm were stained with the following antibodies: Ki67 (Cell Signaling Technology, 12202), P-S5 PolII (Abcam, ab193467), P-S2 PolII (Abcam, ab5095), and P-T161/T160 CDK1/CDK2 (Abcam, ab201008). Staining was visualised using Dako REAL DAB + Chromogen (Agilent Technologies). Slides were counterstained with haematoxylin. Staining was quantified in a blinded fashion in two different fields of view per tumour in three animals per group.

### Statistical analyses

Statistical analyses were carried out using GraphPad Prism v9.0. Pairwise comparisons were performed using the Student’s *t* test. Multiple comparisons were carried out using one-way or two-way analysis of variance followed by Dunnett’s or Šídák’s multiple comparisons test unless otherwise stated. In vivo tumour growth rate was defined as the slope of the linear regression described by tumour volume data points over time. All experiments were conducted with at least two biological repeats. Significant *p* values are displayed as follows: *p < 0.05, **p < 0.01, ***p < 0.001, ****p < 0.0001. Where no asterisks are displayed, no significant differences were found.

### RNA-sequencing and analysis

Total RNA was extracted from 30 mg frozen tumour using the Monarch Total RNA Miniprep Kit (NEB). RNA library preparation and sequencing was performed by Novogene (UK) using 1 µg total RNA. Briefly, sequencing libraries were generated using NEBNext Ultra RNA Library Prep Kit for Illumina (NEB). Library quality was assessed using the Ilumina Bioanalyser 2100 system (Agilent Technologies). The library preparations were sequenced on a NovaSeq 6000 System (Illumina) and paired-end reads were generated. Clean reads were obtained from raw reads in FASTQ format by removing adaptors, poly-N sequences, and low-quality reads. Paired-end clean reads were aligned to the reference genome (GRCh38) using STAR v2.5 software. HTSeq v0.6.1 was used to count the read numbers mapped of each gene. Raw sequencing data and a matrix containing raw counts were submitted to the GEO repository (accession number GSE198488).

### Differential gene expression analysis

Differential expression analysis was performed using the DESeq2 R package (v1.28.1) [46]. Statistical significance was computed using the likelihood ratio test and the resulting *P* values were adjusted using the Benjamini and Hochberg’s approach. Genes with an adjusted *p* value (padj) <0.05 were assigned as differentially expressed. Gene clustering was performed using the degPatterns clustering tool from the DEGreport package (v1.24.1) [47].

### Gene set enrichment analysis

Gene set enrichment analysis (GSEA) was performed using GSEA 4.1.0 software (Broad Institute) using the normalised counts table estimated by DESeq2 for all genes and the Hallmark collection of gene sets (Molecular Signatures Database) [48]. Statistical significance was computed using 10,000 gene set permutations and the analysis was run using default parameters. The gene sets with false discovery rate (FDR) < 0.1 were considered statistically significantly enriched.

## Results

### CT7001 target engagement in LNCaP prostate cells

Previous in vitro kinase assays showed CT7001 selectively targets CDK7, although higher concentrations additionally inhibited the activities of other CDKs [30]. Transitioning from biochemical to cellular environments may alter target activity and selectivity (e.g. due to differences in protein structure and accessibility or due to off-target binding). Therefore, we carried out cellular thermal shift assays (CeTSA) in intact LNCaP cells to investigate which CDK targets are engaged by CT7001 in a cellular model with relevance to prostate cancer. In this assay, binding of a drug to a target leads to formation of a drug–target complex with shifted heat-stability relative to the unbound target; as a result, the amount of soluble target increases in a dose-dependent manner following heat shock and can be determined by immunoblotting [39, 49].

**Fig. 1:**
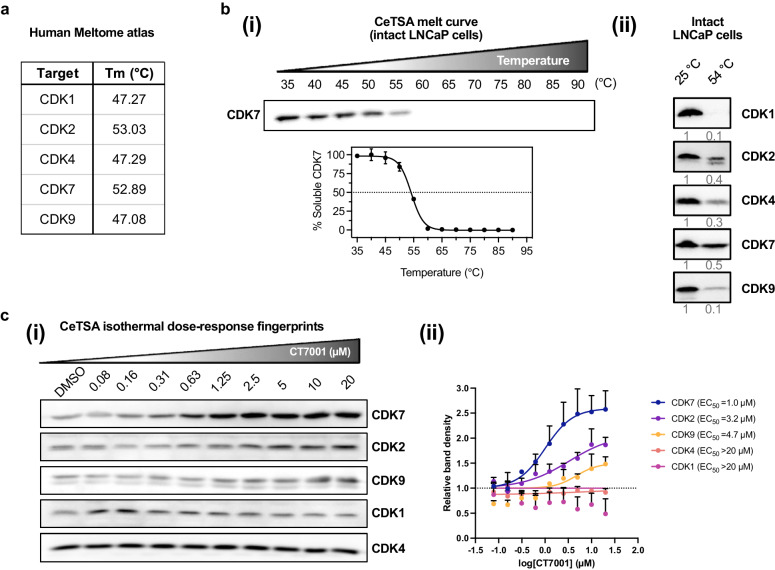
CT7001 target engagement in live LNCaP prostate cells. **a** Calculated melting temperatures (*T*_m_) of human CDK targets. **b** (i) Validation of the CDK7 melt curve in live LNCaP cells and (ii) immunoblots showing the effect of heat shock (54 °C) on soluble levels of CDK1, CDK2, CDK4, CDK7, and CDK9. Numbers underneath blots represent remaining fraction of soluble protein. **c** (i) Cellular thermal shift assay (CeTSA) isothermal dose-response fingerprints at 54 °C in intact LNCaP cells treated with the indicated CT7001 concentrations for 3 h and (ii) quantification of target engagement relative to untreated samples. All immunoblots are representative of 3 biologically independent experiments.

To determine the melting characteristics for putative human CDK targets of CT7001, thermal proteome stability data for human cell lines were downloaded from the Meltome atlas [38]. The thermal profiles of CDK1, -2, -4, -7, and -9 were used to interpolate melting temperatures, *T*_m_, defined as the temperature which aggregates 50% soluble protein. The T_m_ of the different CDKs ranged between ~47 and 53 °C (Fig. 1a). The Meltome data for CDK7 were validated in LNCaP cells (Fig. 1b(i)). We next confirmed that heating of live LNCaP prostate cells at 54 °C for 3 min decreases soluble CDK levels sufficiently to allow exploration of target engagement within the same cell suspension (Fig. 1b(ii)). CeTSA isothermal dose-response fingerprints were then generated at 54 °C using intact LNCaP cells treated with increasing concentrations of CT7001 (0–20 μM) for 3 h. Stabilisation of CDK7, CDK2, and CDK9, but not of CDK4 or CDK1, was observed, as illustrated by increased protein levels compared with DMSO treatment (Fig. 1c(i)). Additionally, we noted earlier engagement with CDK7, consistent with the compound’s reported selectivity (Fig. 1c(ii)) [30]. Overall, these data suggest that CT7001 can bind preferentially to CDK7 but has a degree of engagement also with CDK2 and CDK9 at higher concentrations, which could contribute to efficacy in preclinical models.

### CT7001 inhibits cell growth and promotes cell cycle arrest in vitro

**Fig. 2:**
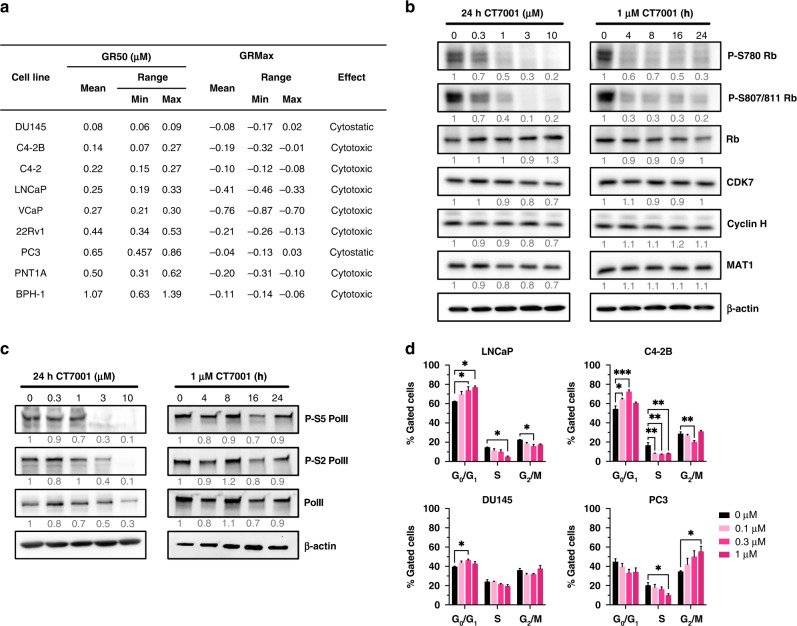
CT7001 inhibits proliferation of prostate cell lines and disrupts cell cycle progression. **a** Table summarising growth rate (GR) metrics in prostate lines treated with CT7001 for 72 h (*n* = 3–4 per cell line). **b** Immunoblots from LNCaP cells treated with CT7001 showing effect on Rb phosphorylation and CAK expression (representative of *n* = 3). **c** Immunoblots from LNCaP cells treated with CT7001 showing effect on PolII phosphorylation (representative of *n* = 3). Numbers underneath blots represent relative band density quantified across 3 independent repeats. Data were adjusted to the loading control (β-actin). **d** FACS analysis of prostate cancer cell lines treated with CT7001 for 72 h and stained with propidium iodide showing cell cycle distributions (*n* = 3). Data are presented as mean ± SEM. *p* values were determined using one-way ANOVA followed by Dunnett’s multiple comparisons test.

The ability of CT7001 to inhibit growth of prostate cancer cell lines was evaluated using growth rate (GR) inhibition studies. GR curves and associated GR metrics are not confounded by the number of cell divisions taking place over the course of the experiment (as opposed to percent viability curves and traditional drug sensitivity metrics) [42], and thus represent an improved methodology to measure drug sensitivity and have been proposed as replacements for conventional metrics [50]. Dose-dependent GR inhibition was observed in all cell lines treated with CT7001 over 72 h (Supplementary Fig. S1A). We calculated the drug concentration that reduces GR by 50% (GR_50_, the primary GR metric for drug potency) as well as the maximal measured GR value (GR_Max_, the primary GR metric for drug efficacy), and summarised these values for each prostate line tested in Fig. 2a. GR_50_ ranged between 0.08 and 0.65 µM for malignant prostate cell lines, suggesting potent growth inhibition consistent with selective engagement with CDK7 based on CeTSA (Fig. 1), while negative GR_Max_ values were indicative of cytotoxicity in all lines except PC3 and DU145. GR_50_ concentrations for the two non-malignant cell lines (BPH-1 and PNT1A) were respectively 3.5-fold and 1.7-fold higher than the average GR_50_ for malignant lines. In LNCaP cells, inhibition of CDK7 activity was associated with markedly reduced retinoblastoma (Rb) phosphorylation, a downstream target of cell cycle CDKs, while levels of CDK7, cyclin H and MAT1 were unaffected (Fig. 2b). In contrast, decreased PolII CTD phosphorylation at S5 (a direct substrate of CDK7) and at S2 (phosphorylation mediated by CDK9) was only observed at concentrations above the GR_50_ of prostate cell lines and above the CDK7 CeTSA EC_50_, respectively (Fig. 2c). This indicates that low but growth-inhibitory concentrations of CT7001 are sufficient to suppress the Rb-pathway and prohibit cell proliferation but are likely insufficient to cause global PolII transcription inhibition. Cell cycle analysis of asynchronous cell populations treated with CT7001 and stained with propidium iodide showed significant reduction of cells in S phase, concomitantly with an increase in diploid (G_0_/G_1_) cells in LNCaP, C4-2B, and, to a lesser extent, in DU145 cells, consistent with Rb inhibition, and an increase in tetraploid (G_2_/M) cells in PC3 cells (Fig. 2d and Supplementary Fig. S1C). The G_2_/M arrest observed in PC3 cells after CT7001 treatment suggests this line carries defects in G_1_/S or S phase checkpoints which alter response to treatment, while a dampened response in DU145 cells might be related to the Rb-deficiency reported for this cell line [51]. Despite these differences, all four cell lines showed a decrease in the proportion of cells in the S phase. Mitosis markers (phosphorylated Rb, CDK1/CDK2, and histone H3) were decreased in LNCaP and PC3 cells, confirming a reduction in the fraction of proliferative cells (Supplementary Fig. S1B). These effects on cell cycle and proliferation are similar to those reported for the covalent CDK7-selective inhibitor YKL-5-124 [21]. Collectively, these data support that prostate cancer cell growth and cell cycle progression are substantially disrupted by CT7001 treatment.

### CT7001 triggers activation of the p53 tumour suppressor pathway

**Fig. 3:**
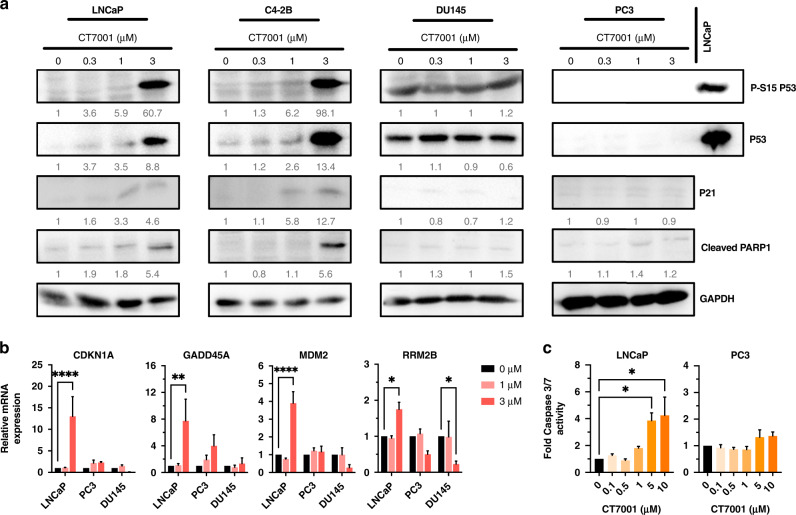
CT7001 induces activation of the p53 tumour suppressor and promotes apoptosis. **a** Immunoblots from prostate cancer cell lines treated with CT7001 for 48 h (representative of *n* = 3). Numbers underneath blots represent relative band density quantified across 3 independent repeats. Data were adjusted to the loading control (GAPDH). For PC3 blot, LNCaP extract was included as a positive control. **b** RT-qPCR analysis of p53 gene targets in LNCaP, PC3, and DU145 cells treated with CT7001 for 24 h (*n* = 3). *p* values were determined using two-way ANOVA followed by Šídák’s multiple comparisons test. **c** Induction of apoptosis monitored via caspase 3/7 assays in LNCaP and PC3 cells following treatment with CT7001 for 72 h (*n* = 3). *p* values were determined using one-way ANOVA followed by Dunnett’s multiple comparisons test. Data are presented as mean ± SEM.

A cytotoxic tendency was observed in GR studies in most prostate lines except DU145 and PC3 cells, which are p53-deficient. This prompted us to investigate whether CT7001 causes activation of the p53 pathway. Indeed, accumulation of p53 protein and increased serine-15 (S15) phosphorylation occurred in p53-intact LNCaP and C4-2B cells after 48 h of treatment, but not in loss-of-function p53-mutant DU145 cells or PC3 cells (Fig. 3a). Activation of p53 was most obvious following treatment with CT7001 concentrations over 1 µM and was concomitant with increased levels of cleaved PARP1, a marker of apoptosis, and of p21, a CDK inhibitor which promotes cell cycle arrest. Further, transcriptional activation of known p53 target genes was confirmed in LNCaP cells but not in PC3 or DU145 cells (Fig. 3b) and caspase 3/7 assays confirmed a significant increase in apoptosis in LNCaP cells, but not in PC3 cells, upon treatment with CT7001 concentrations up to 10 µM (Fig. 3c). These data indicate activation of p53 signalling contributes to cell cycle arrest and apoptosis in response to CT7001 treatment.

### CT7001 impairs androgen-induced transactivation of AR in vitro

**Fig. 4:**
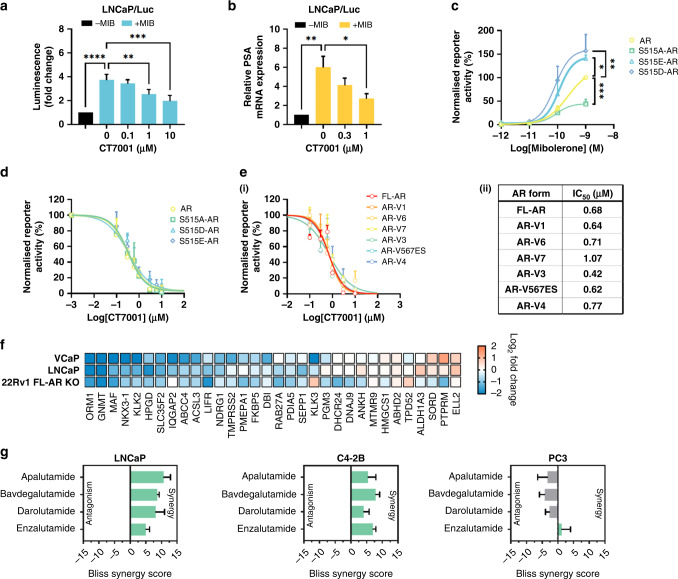
CT7001 interferes with AR transactivation. **a** Luciferase assay in LNCaP/Luc cells treated with CT7001 in the presence of 1 nM mibolerone (MIB) for 24 h (*n* = 6). Data are presented as fold change relative to no androgen control. **b** PSA mRNA expression in LNCaP/Luc cells treated as in **a** (*n* = 3). **c** AR reporter activity in COS-1 cells transfected with wild-type or S515 mutant AR plasmids. Cells were treated for 24 h. Responses were normalised to β-galactosidase for transfection efficiency. Data are presented as % activity relative to wild-type AR, with the highest response representing 100% activity (*n* = 4–5). **d** AR reporter activity in COS-1 cells transfected as in **c** and treated with CT7001 for 24 h in the presence of 1 nM MIB (*n* = 3–4). **e** (i) AR reporter activity in COS-1 cells transfected with full-length AR (FL-AR) or AR variants (AR-V) plasmids. Transcriptional repression by CT7001 was assessed after 24 h in the presence of 1 nM MIB (*n* = 3–4). (ii) Table summarising AR reporter activity IC_50_ for CT7001 treatment of FL-AR and AR-V. **f** Gene expression (RT-qPCR) of 32 AR targets in prostate lines. Cells in presence of androgen were treated ±1 µM CT7001 for 24 h, the colour represents fold change in presence vs absence of CT7001 (*n* = 2). **g** Bliss synergy scores for CT7001 combinations with AR-targeting compounds (*n* = 4). Data are presented as mean ± SEM. *p* values in **a**–**c** were determined using one-way ANOVA followed by Dunnett’s multiple comparisons test.

Given previous reports suggesting a coactivator role of CDK7 in AR-dependent transcription [23, 25], we sought to explore the ability of CT7001 to inhibit AR transactivation. We used the LNCaP/Luc cell line, which has endogenous expression of an active mutant (T877A) form of AR, and an integrated androgen-responsive luciferase reporter [37]. Androgen treatment (mibolerone) induced AR reporter activity, which was suppressed in a concentration-dependent manner by CT7001 (Fig. 4a). Furthermore, mRNA expression of the endogenous AR target gene PSA also decreased in response to CT7001 treatment (Fig. 4b). This effect was not due to reduced AR levels (Supplementary Fig. S2A), impaired AR nuclear translocation in response to ligand (Supplementary Fig. S2B, C), or reduced AR chromatin binding at well-characterised androgen response elements (Supplementary Fig. S2D).

To investigate whether the mechanism of AR repression involves the reported phosphorylation of AR by CDK7 at S515, we employed AR luciferase reporter assays in COS-1 cells transfected with expression vector encoding either wild-type AR or AR in which S515 was replaced either by non-phosphorylatable alanine (S515A-AR), or by the phosphomimetic aspartic acid (S515D-AR) or glutamic acid (S515E-AR). Cells were co-transfected with an androgen-responsive luciferase reporter and β-galactosidase reporter as internal transfection efficiency control. Androgen treatment stimulated the transcriptional activity of wild-type AR and mutant AR proteins in a concentration-dependent manner (Fig. 4c). Relative transcriptional activity was lower than wild-type AR for the S515A-AR mutant and greater for the S515E-AR and S515D-AR mutants, consistent with previous reports [24, 25, 52]. We reasoned that if decreased phosphorylation of AR at S515 is essential for the inhibitory activity of CT7001, then the S515E and S515D substitutions would abrogate its effect. However, treatment with CT7001 suppressed transactivation of wild-type and S515 AR mutants with similar potency and efficacy (Fig. 4d), suggesting CT7001 suppresses AR-driven transcription via a different mechanism.

We also sought to investigate whether AR repression in response to CT7001 treatment requires a functional AR LDB. Similar reporter assays were carried out in COS-1 cells using plasmids encoding full-length AR (FL-AR) or constitutively active AR-Vs that lack the LBD (depicted in Supplementary Fig. S2E) which are insensitive to androgen treatment (Supplementary Fig. S2F), and for which there are no approved inhibitory compounds. Treatment with CT7001 repressed transcription mediated by FL-AR with EC_50_ of 0.68 µM, and by all AR-Vs tested, with EC_50_ ranging between 0.42 and 1.07 µM (Fig. 4e). To validate our findings from AR luciferase reporter assays and investigate transcriptional effects in further prostate cancer cell lines, we measured transcript levels of 32 known AR target genes, comprising an AR activity signature (a list of TaqMan qPCR assays is provided in Additional File 2, Supplementary Table 2). In LNCaP cells, which express FL-AR, and VCaP cells, which express both FL-AR and AR-V, treatment with 1 µM CT7001 repressed expression of most AR targets (Fig. 4f). We repeated this experiment in the 22Rv1 FL-AR KO cell line, which completely lacks FL-AR and in which expression of AR targets is maintained solely by constitutively active AR-Vs [35]. As in the other cell lines, treatment with 1 µM CT7001 was sufficient to decrease the expression of most AR target genes measured (Fig. 4f). The results from AR reporter assays and gene expression analyses of AR target genes in prostate cancer cell lines support the hypothesis that CT7001 represses transcription mediated by both full-length AR and truncated, constitutively active AR-Vs in vitro. Collectively, these indicate AR pathway suppression as a secondary mode of action of CT7001 in prostate cancer cells, in addition to cell cycle inhibition. Thus, we next explored potential synergistic effects on cell growth between CT7001 and four AR-targeted compounds: the second-generation antiandrogens apalutamide, darolutamide, and enzalutamide, and the AR-specific protein degrader bavdegalutamide/ARV-110. Co-treatment with CT7001 and AR-targeted therapies resulted in additive-to-synergistic growth inhibition in the AR-positive hormone-dependent LNCaP and CRPC C4-2B cell lines, as indicated by positive Bliss independence scores (Fig. 4g and Supplementary Figs. S3 and S4). The combinations were, as expected, not synergistic in the AR-negative CRPC PC3 cell line.

### CT7001 and enzalutamide suppress tumour growth in vivo in an additive manner

**Fig. 5:**
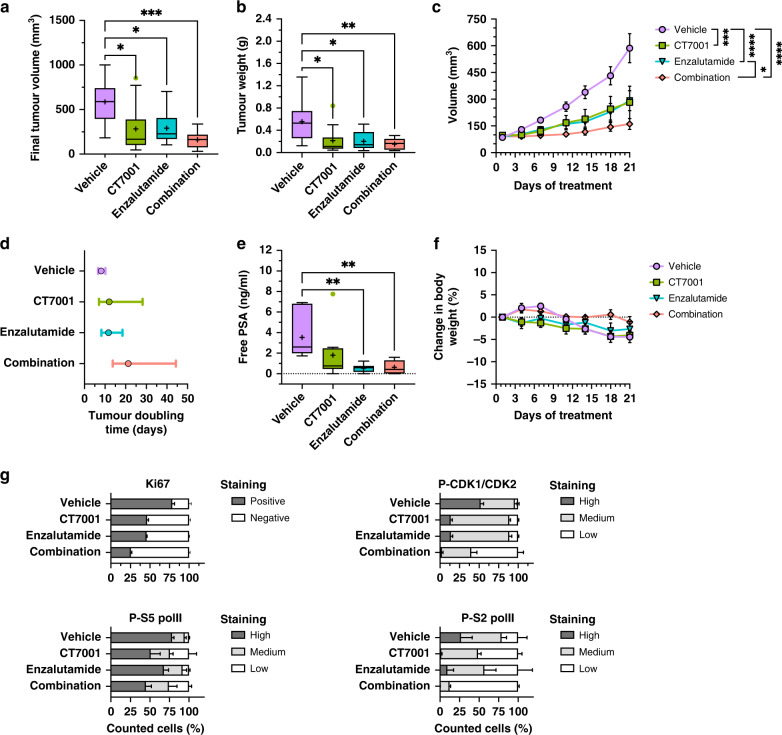
CT7001 administration prevents CRPC growth in vivo and has additive effects in combination with enzalutamide. **a** Tumour volumes on day 21 for male NSG mice bearing C4-2B xenografts following daily oral treatment with vehicle, CT7001 (50 mg/kg), enzalutamide (25 mg/kg) or combination (*n* = 10 per group). **b** Resected tumour weights at the end of 21 days of treatment. **c** Mean tumour volumes ± SEM over the course of treatment. Linear regression showed statistically significant differences in growth between different treatment groups. **d** Tumour doubling times ± 95% confidence intervals. **e** Plasma free prostate-specific antigen (PSA) levels in treated mice. **f** Mean change in body weight ± SEM of treated mice over the course of 21 days of treatment. **g** Quantification of immunohistochemistry (representative images in Supplemental Fig. 5D) staining in *n* = 3 animals per group (mean ± SEM). *p* values were determined using one-way ANOVA followed by Šídák’s multiple comparisons test. Data in **a**, **b**, **e** are presented using Tukey boxplots. Mean values are plotted as a “+” and outliers are shown as individual data points. *p < 0.05; **p < 0.01; ***p < 0.001; ****p < 0.0001.

To assess tumour growth inhibition of CT7001 alone or in combination with enzalutamide, a widely used antiandrogen, immunocompromised (NSG) male mice with established subcutaneous C4-2B xenografts were assigned into 4 treatment groups: vehicle, CT7001 (50 mg/kg) alone, enzalutamide (25 mg/kg) alone, or a combination of enzalutamide and CT7001. On day 21 of daily oral treatment, CT7001 and enzalutamide monotherapy reduced final tumour volume by 52% and 50% respectively, while combination therapy reduced final tumour volume by 73%, compared to vehicle treatment (Fig. 5a). A significant reduction in the weights of excised tumours was also evident for all treatments (Fig. 5b). Regression analysis using longitudinal tumour volume data showed a significant reduction in growth rate in all treatment groups compared to vehicle (vehicle vs. CT7001 *p* = 0.0006; vehicle vs. enzalutamide *p* < 0.0001; vehicle vs. combination *p* < 0.0001) and, additionally, a significant reduction in tumour growth rate in the combination arm compared to enzalutamide alone (*p* = 0.0156) but no significant difference between the growth-rates of CT7001-treated and combination-treated tumours (*p* = 0.3145) (Fig. 5c). Mean tumour doubling time was 8 days for vehicle-treated mice, 12 days for CT7001-treated mice, 11 days for enzalutamide-treated mice, and 21 days for mice treated with both CT7001 and enzalutamide (Fig. 5d). Tumour growth inhibition was also associated with significantly reduced plasma free PSA in enzalutamide- and combination-treated mice relative to vehicle controls, and with a similar trend in CT7001-treated mice (Fig. 5e). All treatments were generally well-tolerated, with less than 5% body weight loss on average in all treatment arms, which was comparable to the weight loss observed in vehicle controls (Fig. 5f). Additionally, plasma AST and urea levels appeared unchanged, except for a small reduction in plasma urea concentration in enzalutamide-treated mice relative to vehicle controls (Supplementary Fig. S5A, B). There were no obvious histopathological changes in the liver of treated animals (Supplementary Fig. S5C). Immunohistochemistry of resected tumours showed CT7001 monotherapy reduced Ki67 expression, with further decrease observed in tumours treated with a combination of enzalutamide and CT7001 (Fig. 5g and Supplementary Fig. S5D). In addition, T-loop phosphorylation of CDK1/CDK2 was additively decreased by CT7001 and enzalutamide treatments, and a decrease in PolII phosphorylation was detected in tumours treated with CT7001 alone or in combination with enzalutamide.

**Fig. 6:**
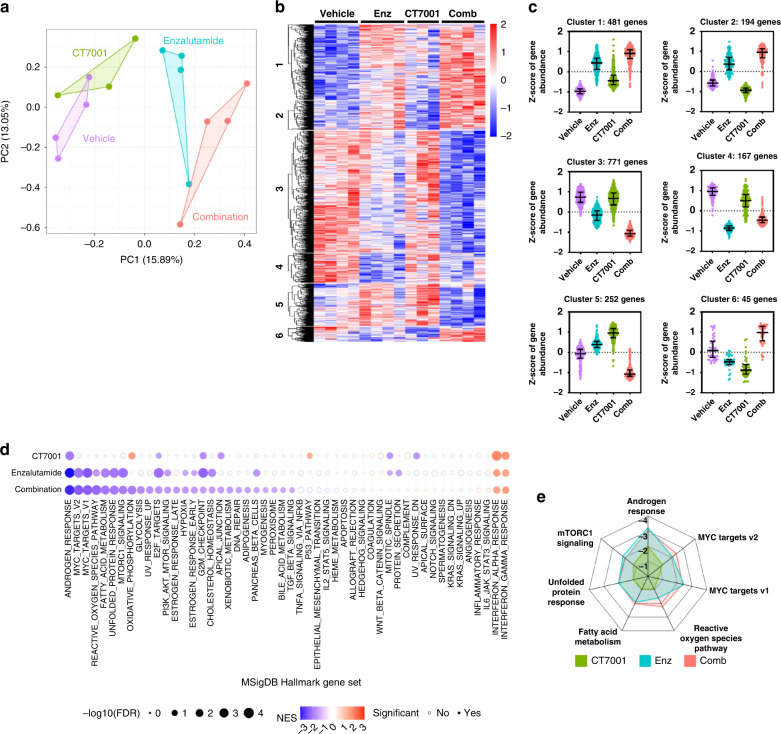
Transcriptomic analysis of C4-2B xenografts. **a** Unsupervised principal component analysis (PCA) clustering of treated xenografts. **b** Heatmap of differentially expressed genes identified through DESeq2 likelihood ratio test as changing in expression across different treatments. **c** Clustering of differentially expressed genes based on expression profiles across different treatments. **d** Gene set enrichment analysis results showing MSigDB hallmark gene sets significantly enriched in treatment groups vs vehicle. Dot size represents −log10 false discovery rate (FDR) while colour indicates normalised enrichment score (NES). Significantly enriched gene sets (FDR < 0.1) are shown using colour-filled dots. **e** Radar plot displaying NES for CT7001, enzalutamide and combination for the top hallmarks gene sets enriched in the combination group.

Finally, we sought to identify drug-induced transcriptome changes and biological pathways associated with treatment response by carrying out RNA-seq analysis of the treated C4-2B xenografts (*n* = 3–4 per group). Unsupervised principal component analysis (PCA) captures 28.94% of the gene expression variance in the first two PCs and shows separation of the different groups, with the enzalutamide and combination groups clustering furthest from the vehicle group (Fig. 6a). We performed differential gene expression analysis using DESeq2 [46] and computed statistical significance using a likelihood ratio test. This analysis identified 1915 differentially expressed genes that showed significant changes in expression across the four different groups (padj<0.05). Clustering analysis performed using the DEGreport package [47] identified 6 major differential gene expression clusters (Fig. 6b, c). Clusters 1-4, representing a large majority of 1612 genes, showed altered expression in response to enzalutamide and combination treatment, while treatment with CT7001 alone led to only small changes in expression. This pointed to enzalutamide as the major driver of transcriptional effects in response to combination therapy. We performed gene set enrichment analysis (GSEA) using a matrix containing DESeq2-normalised counts for all detectable genes in the different groups and calculated enrichment scores, relative to vehicle, for the Hallmark gene sets collection [48] (Fig. 6d). With respect to CT7001 treatment alone, the results of the GSEA are consistent with the effects of CT7001 treatment observed in vitro. Negative enrichment for several cell cycle-related gene sets (e.g. G2M checkpoint, E2F targets, and mitotic spindle) and the hallmark androgen response, and positive enrichment for the hallmark p53 pathway were noted (Supplementary Figs. S6 and S7). In tumours treated with enzalutamide alone or with a combination of enzalutamide and CT7001, GSEA identified negative enrichment of several gene sets, including the hallmark androgen response, MYC targets, reactive oxygen species and fatty acid metabolism (Fig. 6d). Reassuringly, the top negatively regulated gene set for enzalutamide and combination treatments was the hallmark androgen response; interestingly, this was also the case for CT7001 alone. A few hallmark gene sets were exclusively negatively enriched in the combination group (e.g. oxidative phosphorylation, glycolysis, oestrogen responses, DNA repair). However, the top gene sets enriched in the combination group substantially overlapped with those enriched in the enzalutamide group (Fig. 6e). This indicates that addition of CT7001 to enzalutamide at therapeutic doses contributes little to the transcriptional effect of enzalutamide and suggests cell cycle inhibition by CT7001 as the primary mechanism underpinning the additional growth repression observed in response to combination therapy.

## Discussion

In this report, we present a systematic assessment of the mechanism of action and preclinical efficacy of CT7001 as a single agent or combined with the second-generation antiandrogen enzalutamide in established preclinical models of CRPC. Using CeTSA in LNCaP cells, we show CT7001 preferentially targets CDK7 over CDK2 and CDK9. This complements published studies using cell-free in vitro kinase assays [30] and confirms CT7001’s potent and selective pharmacology against CDK7 in living prostate cancer cells. In contrast to one report suggesting CDK4 as a target of CT7001 [53], we found no evidence of direct engagement with CDK4 or with CDK1 at concentrations up to 20 µM.

Treatment with CT7001 induces potent growth inhibition and promotes cell cycle arrest in prostate cancer lines with submicromolar potency, while higher drug concentrations can induce activation of the p53 pathway and promote apoptosis. Our results show that CT7001 also inhibits the transcriptional activity of AR, a key transcription factor and oncogenic driver in prostate cancer. Increased sensitivity of AR-positive (compared to AR-negative) prostate cancer cell lines to THZ1, an inhibitor of CDK7/CDK12/CDK13, has been previously reported [23]. With respect to CT7001, potent growth inhibition was achieved in both AR-positive (LNCaP, C4-2, C4-2B, VCaP and 22Rv1) and AR-negative (DU145, PC3) lines.

Notwithstanding, complete loss of AR expression is rare in CRPC, where reactivation of AR signalling drives resistance to androgen deprivation therapy. This frequently involves mutation or loss of the AR LBD. In this report, we demonstrate that CT7001 treatment successfully inhibits growth and AR-dependent transcriptomes of cell lines modelling these scenarios: LNCaP cells carry a point mutation in the AR LBD (T877A) which confers promiscuous activation by alternative ligands, VCaP cells display AR gene amplification in addition to expressing constitutively active AR-Vs, while the engineered 22Rv1 FL-AR KO line displays ligand-independent AR transcriptome driven by AR-Vs and intrinsic resistance to AR-targeting therapies. Direct inhibition of ectopically expressed AR-Vs was also demonstrated. As CT7001 mechanistically functions independently of the AR LBD, unlike all approved AR-targeted compounds, it is a good therapeutic candidate for CRPC tumours with AR reactivation. We also explored the therapeutic potential of combining CDK7 inhibition with the widely prescribed antiandrogen enzalutamide and found additive tumour growth repression in vivo, likely mediated largely through CT7001’s potent repression of cell cycle progression. Further research is warranted to determine whether, in models displaying resistance to antiandrogens, AR pathway suppression by CT7001 becomes essential for therapeutic efficacy.

Considering the more widespread effects of CDK7 on PolII-dependent transcription, mounting evidence suggests targeting transcriptional kinases using selective inhibitors could provide a sufficient therapeutic window to be effective without the initially feared toxicity [54, 55]. Our data indicate that, while CT7001 treatment can reduce PolII phosphorylation, this effect only occurred at concentrations >10-fold greater than the GR_50_ in LNCaP prostate cancer cells. Therefore, inhibition of the Rb and AR pathways may be sufficient to explain the growth inhibition observed at lower concentrations. This is in line with recent work using the CDK7-selective covalent inhibitor YKL-5-124, which caused no change in PolII phosphorylation at growth-inhibitory doses [21, 22], while the CDK7/12/13 inhibitor THZ1 decreases PolII phosphorylation at nanomolar concentrations [56]. However, it is worth mentioning that a reduction in PolII phosphorylation occurred in vivo following treatment with CT7001 and that a change in the steady-state mRNA levels resulting from global repression of transcription would not be evident in the generated RNA-seq dataset. Therefore, we cannot rule out the possibility that inhibition of PolII transcription contributed to the efficacy observed in vivo.

Several questions remain regarding the mechanism of action of CT7001 in prostate cancer. Firstly, activation of p53 presumably contributes to CT7001-induced apoptosis. The highly metastatic PC3 cell line, which does not express p53 protein, appears resistant to apoptosis in response to CT7001 treatment. In addition, activation of p53 transcriptional programme has been demonstrated to sensitise some cancer cells to CDK7 inhibition by promoting pro-apoptotic pathways [57]. As the p53 tumour suppressor is frequently mutated in prostate cancer and has prognostic significance [4, 58], future studies should explore whether enhanced sensitivity or/and induction of apoptosis in response to CT7001 treatment is dependent on an intact p53 pathway. Indeed, preliminary data from the clinical trial strongly indicate that *TP53* status is associated with response to CT7001 therapy in patients with metastatic hormone receptor-positive/HER2-negative breast cancer [34]. Secondly, in addition to suppressing cell proliferation, CDK7 inhibition by YLK-5-124 was found to induce interferon gamma signalling along with other inflammatory response pathways in models of small cell lung cancer [22]. This, in turn, triggered robust immune cell signalling, which potentiated the antitumour immune response and enhanced response to anti-PD-1 immunotherapy. Whether CT7001 treatment can provoke similar antitumour immune responses in immunocompetent murine models of prostate cancer remains to be explored.

In summary, the data presented here demonstrate that the orally bioavailable CDK7 inhibitor CT7001 impedes malignant cell growth by targeting proliferation pathways and, in addition, downregulates oncogenic AR signalling and induces apoptosis in CRPC models. This multifaceted mechanism of action drives additive combinatorial activity with the antiandrogen enzalutamide in the C4-2B xenograft model of advanced CRPC. Therefore, this study supports CDK7 inhibition as a therapeutic strategy for CRPC and provides a rationale for new combination regimens consisting of CDK7 inhibitors and antiandrogen therapy, all the more so as CT7001 demonstrated acceptable safety profile with evidence of target engagement in clinical trials.

### Supplementary information

Supplemental Figures

Supplemental Methods

ARRIVE checklist

## Data Availability

The RNA-sequencing data generated in this study were deposited in NCBI Gene Expression Omnibus (GEO accession GSE198488). The melting curves for CDK proteins were downloaded from the online Meltome Atlas (http://meltomeatlas.proteomics.wzw.tum.de:5003/). All other data generated or analysed during this study are included in this article and its supplementary information provided in Additional file 1 and Additional file 2.
