# Correction: The CDK7 inhibitor CT7001 (Samuraciclib) targets proliferation pathways to inhibit advanced prostate cancer

**DOI:** 10.1038/s41416-024-02606-w

**Published:** 2024-02-14

**Authors:** Theodora A. Constantin, Anabel Varela-Carver, Kyle K. Greenland, Gilberto Serrano de Almeida, Ellen Olden, Lucy Penfold, Simon Ang, Alice Ormrod, Damien A. Leach, Chun-Fui Lai, Edward K. Ainscow, Ash K. Bahl, David Carling, Matthew J. Fuchter, Simak Ali, Charlotte L. Bevan

**Affiliations:** 1 Imperial Centre for Translational and Experimental Medicine, Department of Surgery and Cancer, Imperial College London, Hammersmith Hospital Campus, London, UK; 2 MRC London Institute of Medical Sciences, Imperial College London, Hammersmith Hospital, London, UK; 3 Carrick Therapeutics, Nova UCD, Bellfield Innovation Park, Dublin, 4 Ireland; 4 Department of Chemistry, Molecular Sciences Research Hub, Imperial College London, White City Campus, London, UK

**Keywords:** Prostate cancer, Molecular biology

Correction to: *British Journal of Cancer* (2023) **128**:2326-2337; 10.1038/s41416-023-02252-8, Article published online 19 April 2023

Following the original publication, the authors were notified of an error present in Figure 1, specifically Figure 1bii, where the same image file was used for CDK4 and CDK7.

The corrected version of Figure 1 is produced here.
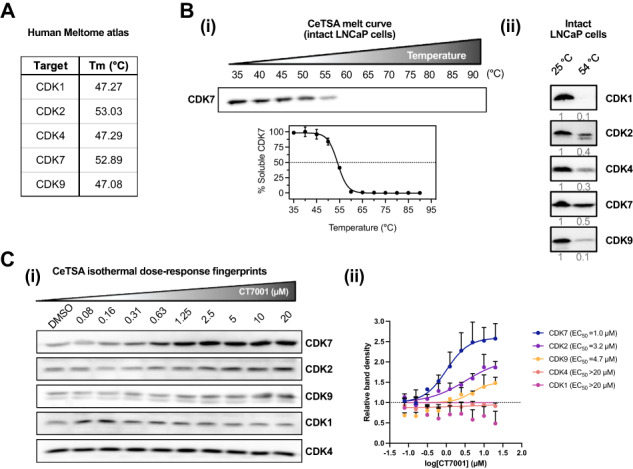


The authors provided the journal with the original data. The correction does not have any effect on the results or conclusions of the paper.

The original article has been corrected.

